# Transesophageal Echocardiography in Transcatheter Mitral Valve Replacement

**DOI:** 10.3390/jcm14227966

**Published:** 2025-11-10

**Authors:** Beatrice Bacchi, Kendra Derry, Tasnim Vira, Sami Alnasser, Paola Keese Montanhesi, Neil Fam, Gianluigi Bisleri

**Affiliations:** 1 Division of Cardiac Surgery, St. Michael’s Hospital, University of Toronto, 36 Queen Street E, Toronto, ON M5B 1W8, Canada; beatricebacc@gmail.com (B.B.); paolakeese@gmail.com (P.K.M.); 2 Department of Anesthesiology & Pain Medicine, St. Michael’s Hospital, University of Toronto, 36 Queen Street E, Toronto, ON M5B 1W8, Canada; kendra.derry@unityhealth.to; 3 Terrence Donnelly Heart Center, St. Michael’s Hospital, University of Toronto, 36 Queen Street E, Toronto, ON M5B 1W8, Canada; tasnim.vira@unityhealth.to (T.V.); sami.alnasser@unityhealth.to (S.A.); neil.fam@unityhealth.to (N.F.)

**Keywords:** transesophageal echocardiography, transcatheter mitral valve replacement, multimodality imaging, left ventricular outflow tract obstruction

## Abstract

Transesophageal echocardiography (TEE) has emerged as the pivotal imaging modality in transcatheter mitral valve replacement (TMVR), bridging the gap between anatomical complexity and procedural precision. Unlike any other tool, TEE accompanies the patient journey across all stages of TMVR, from patient assessment and device selection to intraprocedural guidance and post-implant surveillance, providing real-time insights into valve anatomy, hemodynamics, and complications. This review consolidates the most recent consensus statements, quantitative thresholds, and device-specific considerations, while also highlighting evolving technologies. By outlining best practices for integrating TEE within multimodality workflows and the Heart Team paradigm, this article offers clinicians a comprehensive and practice-oriented roadmap for optimizing TMVR outcomes.

## 1. Introduction

Transcatheter mitral valve replacement (TMVR) has rapidly evolved from an investigational concept to a viable therapy for selected patients with severe mitral valve disease [1,2,3]. With growing experience and increasingly complex anatomy, the demand for standardized and advanced imaging workflows has become essential.

Imaging, particularly transesophageal echocardiography (TEE), plays a central role throughout the TMVR pathway, from patient selection and procedural planning to real-time guidance and follow-up, allowing for the detection of acute complications and prosthetic dysfunction [3,4]. TEE provides high-resolution, real-time visualization of the mitral valve apparatus, including 3D en-face views critical for device deployment, and enables immediate hemodynamic assessment such as transmitral gradients and left ventricular outflow tract (LVOT) dynamics [5,6,7].

While TEE provides real-time anatomic and functional insights critical for the safety and success of TMVR [8], computed tomography (CT) is the reference for annular sizing and neo-LVOT prediction [9]. Recent EACVI/ASE consensus statements endorse this complementary multimodality approach, with CT as the gold standard for pre-procedural planning and 3D-TEE as the main modality for intraprocedural guidance and hemodynamic assessment [10].

This state-of-the-art review synthesizes consensus recommendations, quantitative thresholds, and emerging TEE technologies relevant to TMVR, with emphasis on patient selection, procedural planning, intraprocedural/postprocedural monitoring, and workflow optimization within the multidisciplinary heart team. By consolidating evidence and expert perspectives, the review aims to provide a comprehensive reference for clinicians engaged in TMVR.

## 2. The Role of TEE in Patient Selection

TEE is pivotal in determining candidacy for TMVR, confirming ineligibility for other transcatheter options (e.g., TEER) and defining the underlying mechanism of valve dysfunction. A thorough pre-procedural echocardiographic assessment guides device choice and anticipates complications. Although TMVR is primarily used for severe mitral regurgitation, the clinical spectrum extends to mitral stenosis, extensive annular calcification, and prior surgical or transcatheter mitral interventions, such as valve-in-valve (ViV) or valve-in-ring (ViR) procedures [4,6]. In this heterogeneous population, TEE provides anatomical and functional insights for candidacy and procedural strategy [11].

### 2.1. TEE Evaluation of the Mitral Apparatus

Assessment should start with a comprehensive analysis of the mitral valve apparatus, including annulus, leaflets, commissures, and subvalvular structures. High-resolution 3D TEE enables detailed anatomical visualization and supports the identification of features that may increase procedural complexity, such as bulky calcification, restricted leaflet motion, or distorted geometry following previous interventions [11,12].

Mitral annular sizing directly influences the prosthesis choice and risks of malposition, paravalvular leak, and device embolization. 3D TEE captures the dynamic-shaped configuration throughout the cardiac cycle with measurements typically indexed to end-systole, when annular dimensions are maximal.

### 2.2. Anatomical Limitations

Small annuli may preclude the use of certain devices due to the risk of oversizing, malposition, or LVOT obstruction (LVOTO) [13]. For example, self-expanding systems such as the Tendyne (Abbott Structural, Santa Clara, CA, USA) valve, though available in multiple sizes, has a large frame and may be unsuitable for very small annuli [14,15,16]. In Tendyne screening, mitral inter-commissural dimensions <30 mm or >50 mm predicted anatomical unsuitability with a 94.4% negative predictive value, mostly due to LVOTO risk [16].

Similarly, balloon-expandable valves used in valve-in-mitral annulus calcification (ViMAC) procedures, such as the Sapien 3 (Edwards Lifesciences), carry higher oversizing risks in small annuli [17], and cannot be repositioned in cases of LVOTO, limiting their use [18].

Conversely, very large annuli may exceed current device ranges, risking incomplete sealing and paravalvular regurgitation [13]. This affects devices like the Sapien 3 (maximal outer diameter of ~29 mm) [17] and the Intrepid system (Medtronic Inc., Minneapolis, MN, USA) [19]. In contrast, the Tendyne system, with a broader size matrix up to ~50 mm, is often preferred for markedly dilated annuli [14] (Table 1).

### 2.3. Assessment of the LVOT

Assessment of the LVOT is equally critical during pre-procedural screening, as obstruction is associated with significantly higher procedural mortality (34.6% vs. 2.4%) [2]. 3D TEE allows for the accurate measurement of baseline LVOT dimensions, including the anteroposterior diameter, area, and its relationship to the anterior mitral leaflet (AML) and interventricular septum. Measurements are generally acquired in mid-systole, when LVOT dimensions are at their narrowest and most predictive of post-implant obstruction [20].

Prediction of the neo-LVOT, the residual outflow tract area following valve deployment, is a central step in risk stratification. The neo-LVOT is estimated by virtually “placing” the prosthesis in relation to the AML and interventricular septum, then calculating the expected cross-sectional area [21,22]. While cardiac CT remains the gold standard for this simulation, TEE provides complementary information on leaflet motion, septal morphology, and chordal structures that may further narrow the outflow tract. A neo-LVOT < 200 mm^2^ is widely recognized as a high-risk threshold, with values between 200–250 mm^2^ considered borderline and require individualized assessment in the context of patient anatomy and device selection [23].

Several anatomical features increase the risk of obstruction and should be specifically assessed by TEE:

- Anterior mitral leaflet length: elongated AML (>25–28 mm) increases the likelihood of systolic displacement and obstruction [24].
- Septal thickness: basal septal hypertrophy >15 mm exacerbates LVOT narrowing, particularly in small ventricles [25].
- Mitral–aortic angle: an acute angle (<120°) between the mitral annular plane and the aortic root reduces the outflow tract “escape route” and amplifies obstruction risk [26,27].
- Subvalvular apparatus: displaced or hypertrophied papillary muscles and prominent chordae may contribute to narrowing of the outflow tract after valve deployment [28].

TEE can also evaluate dynamic LVOT physiology, including systolic anterior motion (SAM) of the AML, turbulence on color Doppler, and elevated gradients, which may predict vulnerability to obstruction.

### 2.4. Cardiac Function Assessment

Beyond valve-specific anatomy, TEE offers an integrated evaluation of global cardiac function and hemodynamics, which is essential for both procedural feasibility and prognostic assessment. Quantification of left ventricular volumes and ejection fraction helps identify patients at risk of poor tolerance to the hemodynamic changes after TMVR, particularly when LVEF is severely reduced (<30%) [29]. Right ventricular (RV) function should also be assessed using TAPSE (<17 mm abnormal), S′ velocity (<9.5 cm/s abnormal), and fractional area change (<35% abnormal), as severe RV dysfunction or pulmonary artery systolic pressure >60 mmHg correlates with adverse outcomes [30,31].

Left atrial (LA) size and remodeling, expressed by indexed LA volume (>34 mL/m^2^), provide insights into disease chronicity and the risk of post-procedural atrial arrhythmias [32,33]. Careful evaluation of the interatrial septum is equally relevant, as septal thickness, fibrosis, or aneurysmal mobility influence the feasibility and optimal location of the transseptal puncture, an essential step for achieving the correct trajectory during TMVR [34,35]. While TEE provides superior anatomic and spatial resolution, TTE remains more reliable for certain functional parameters, including ventricular performance and pulmonary pressure. Thus, TTE and TEE are complementary, providing an integrated pre-procedural evaluation. This concept of multimodality synergy has also been underscored in other valvular settings—for instance, in atrial functional mitral regurgitation [36]. In this context, it is crucial to combine the diagnostic roles of TTE and TEE in defining anatomy and functional severity, a principle equally applicable to transcatheter mitral interventions. Intraoperatively, TEE remains indispensable for anatomic guidance, though Doppler-based gradients may be limited by beam alignment; integration with pre- or post-procedural TTE data ensures accurate hemodynamic interpretation.

### 2.5. Multimodality Imaging

While CT remains the gold standard for device modeling, 3D TEE provides complementary information, particularly when CT quality is limited by arrhythmia, renal dysfunction, or contrast contraindications [9].

TEE enables the real-time assessment of annular size, leaflet tethering, calcification burden, and subvalvular anatomy.

Strong correlations exist between the 3D TEE and CT measurements of mitral annular area and perimeter (r = 0.88–0.92) [37]. However, CT typically yields slightly larger values for area, perimeter, and septal–lateral diameter, while inter-trigonal distance remains comparable [8]. Cardiac magnetic resonance, although less commonly used, can provide additional functional and tissue characterization when necessary [38].

This multimodality approach, discussed within the multidisciplinary Heart Team, ensures that patient selection is accurate, safe, and tailored to the complexity of individual anatomy [11,39] (Table 2).

## 3. Intra-Procedural Monitoring with TEE

Once TMVR candidacy is confirmed, TEE becomes central to procedural execution, offering continuous, real-time visualization of anatomy and hemodynamics. A structured TEE protocol, including the assessment of annular and subvalvular calcification, interatrial septum and puncture trajectory, device orientation, transmitral and LVOT gradients, pulmonary venous flow, and filling pressures, ensures comprehensive guidance throughout the procedure.

By linking pre-implant anatomical evaluation with post-deployment functional validation, TEE optimizes device positioning, detects complications early, and enhances procedural safety.

### 3.1. TEE in Transseptal Puncture

**Figure 1:**
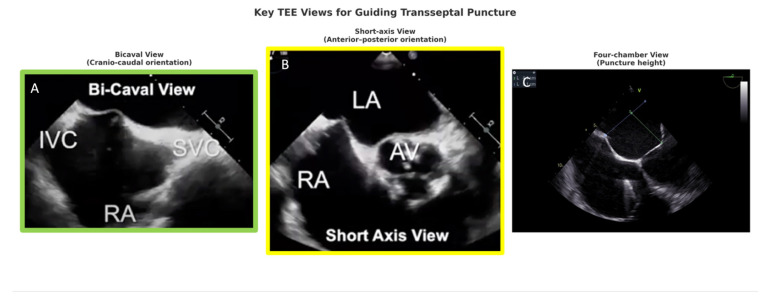
Key TEE Views for Guiding Transseptal Puncture in TMVR. Legend: TEE guidance for transseptal puncture relies on three complementary planes: the bicaval view (**A**) provides cranio–caudal orientation and confirms the puncture site between the superior vena cava (SVC) and inferior vena cava (IVC); the short-axis view at the aortic valve level (**B**) offers anterior–posterior orientation relative to the interatrial septum and aortic valve (AV); and the four-chamber view (**C**) determines puncture height above the fossa ovalis. These orthogonal projections ensure precise and safe transseptal access. LA—left atrium; RA—right atrium.

The transseptal puncture represents one of the most critical determinants of technical success in TMVR. The puncture site and height within the fossa ovalis must provide adequate left atrial workspace and a coaxial trajectory toward the mitral annulus [17]. TEE provides continuous imaging from multiple orthogonal planes:

- Bicaval view: defines superior–inferior orientation and septal tenting,
- Short-axis view (aortic valve level): defines anterior–posterior position, preventing aortic mispuncture,
- Four-chamber view: confirms puncture height above the annular plane (ideally 3.5–4.5 cm) [6,40,41] (Figure 1).

During transseptal puncture, the dedicated needle becomes the key element under TEE guidance. Its tip position and orientation determine the eventual trajectory toward the mitral annulus. Superior punctures generally shift the needle tip anteriorly, whereas inferior punctures result in a more posterior trajectory. Fine adjustments are achieved by rotating the needle: counterclockwise rotation directs the puncture toward the mitral valve plane (‘lose height’), while clockwise rotation moves it away (‘gain height’) [41].

Most TMVR systems require a posterior–mid puncture about 3.5–4.5 cm above the annulus; however, optimal height varies by device design and atrial anatomy [17,41].

This principle becomes particularly relevant when considering the differences among currently available transseptal TMVR systems. Bulky and relatively rigid delivery catheters, such as the Intrepid system (Medtronic, 35 Fr transfemoral–transseptal), generally necessitate a higher and more posterior puncture (~4.0–5.0 cm) to ensure sufficient atrial working space and coaxial alignment, with some reports describing heights exceeding 5 cm in ViV cases [19].

In contrast, balloon-expandable systems like the SAPIEN 3 (Edwards Lifesciences) have a shorter profile and can often be successfully delivered through a lower puncture height (~3.5 cm), provided that coaxiality and device manipulation are maintained [17].

Incorrect puncture location can have significant consequences. Low punctures (<3 cm) create a steep entry angle, predisposing to malalignment or failed deployment; high punctures (>5 cm) reduce coaxiality and maneuverability, especially in patients with large left atria, and may increase tension on the septum. Anterior punctures risk aortic interference, while posterior ones increase septal tension and instability [41,42,43].

Anatomical challenges such as lipomatous septum, aneurysmal interatrial septum, or distortion after prior surgical/percutaneous procedures further complicate transseptal puncture, making meticulous TEE guidance essential to avoid complications such as inadvertent aortic puncture or cardiac tamponade [41,44,45].

### 3.2. Trajectory Planning and Coaxiality

TEE is fundamental not only for guiding transseptal access but also for anticipating and optimizing the trajectory of the delivery system through the left atrium toward the mitral annulus. 3D en-face TEE views provide a surgical perspective of the mitral orifice, allowing operators to verify that the delivery system is coaxial with the annular plane, a prerequisite for stable deployment and complete sealing of the prosthesis [4,6,46,47].

Different TMVR devices have distinct trajectory requirements based on their size, and flexibility. The Tendyne valve, delivered via a large and relatively rigid transapical system (34–36 Fr), demands precise alignment and stable positioning; even minor deviations can complicate deployment due to its bulky double-frame design and apical tether [15,48]. The Intrepid system, also characterized by a bulky, dual-stent structure, requires generous left atrial space to accommodate the delivery sheath and permit smooth orientation of the valve. TEE helps identify patients with small or compressed left atria, where navigation may be challenging [19,49].

In contrast, the Sapien 3 (Edwards Lifesciences) has a more compact and maneuverable delivery system, but an accurate trajectory remains crucial to prevent PVL or device migration. TEE confirms coaxiality, particularly in valve-in-ring or valve-in-MAC cases, where eccentric deployment is common [50,51].

Emerging transseptal systems, such as HighLife (HighLife SAS), are designed to optimize navigation and alignment within the left atrium, though they also require adequate atrial workspace for safe manipulation.

Finally, TEE is essential to identify anatomical barriers, such as septal bulging, prominent ridges, aneurysmal septum, or interatrial thrombus, that may hinder catheter maneuverability or increase the risk of malalignment or procedural complications [52,53].

### 3.3. Calcification and Sealing

TEE provides a meticulous assessment of annular and subvalvular calcification, which can significantly affect prosthesis expansion and sealing. In addition to anatomical imaging, Doppler analysis helps anticipate post-procedural hemodynamic results [3].

High-resolution 3D en-face views allow for the visualization of circumferential calcium and nodular spurs protruding into the left ventricular inflow, features that may incomplete frame expansion, rocking, or PVL [54,55]. While CT remains superior for quantifying calcium burden, TEE uniquely demonstrates the dynamic interaction between calcium and the device, showing whether the sealing skirt achieves full apposition or whether calcific or subvalvular structures impede prosthesis expansion [56].

Among dedicated TMVR devices, the Tendyne valve performs well in patients with extensive or circumferential MAC due to its large sealing cuff and wide size range, providing a stable anchoring zone [47,57,58]. The Intrepid system, with a dual-stent design, can be more sensitive to asymmetric or nodular calcium deposits, which may prevent uniform apposition; however, its conformability is advantageous in anatomies with severe subvalvular calcification or narrow inflow tracts, where a higher, posterior transseptal puncture may improve coaxiality [3,19,59,60].

In ViMAC procedures using Sapien 3, calcium can contribute to device anchoring but also predisposes to under-expansion and eccentric PVL. Real-time TEE is therefore essential to confirm coaxial alignment, full frame expansion, and complete sealing, reducing the risk of malseating or rocking [51,61].

### 3.4. Confirmation of Positioning

Confirming correct valve positioning and deployment is among the most critical intra-procedural uses of TEE as it determines technical success and immediate hemodynamic performance. Real-time 3D-TEE provides en-face “surgical” views of the mitral annulus, allowing for precise assessment of prosthesis orientation and depth before release [3,4,55].

For self-expanding systems such as Tendyne and Intrepid, TEE verifies that the atrial cuff or outer stent frame lies flush with the mitral annular plane, neither protruding excessively into the atrium nor the ventricle. A balanced position with circumferential skirt apposition is confirmed across multiple 3D planes [15,49].

For balloon-expandable systems like Sapien 3, TEE ensures coaxial alignment within the surgical ring or calcified annulus and identifies asymmetric expansion that could lead to embolization or PVL. Deployment depth is device-specific; for Sapien 3, optimal placement positions roughly 70–80% of the frame below the annular plane [3].

### 3.5. Post-Deployment Assessment

After valve release, color Doppler TEE is used immediately to detect paravalvular jets. Even trivial jets can be mapped using 3D color datasets, which precisely identify jet origin (commissural, central, or posterior annulus) and guide additional maneuvers such as balloon post-dilation, repositioning, or percutaneous closure. Persistent or eccentric jets often reflect asymmetric calcification or incomplete frame expansion, findings that can be anticipated from pre-procedural imaging but must be confirmed in real-time by TEE [6,62,63].

Continuous-wave Doppler across the prosthesis provides quantitative assessment of the transmitral gradient. A mean gradient <5 mmHg at 60–80 bpm defines procedural success, while 5–8 mmHg may be acceptable in larger patients or at higher heart rates, but warrants correlation with prosthesis size and LV filling pressures. Gradients >8 mmHg indicate possible underexpansion or malalignment and require immediate evaluation. Underexpanded Sapien 3 valves may respond to additional ballooning, whereas tilted self-expanding devices (Tendyne, Intrepid) may need repositioning or, in rare cases, retrieval and redeployment if feasible [3,49,64,65,66,67].

Pulmonary venous flow patterns add hemodynamic insight: reappearance of dominant systolic forward flow confirms effective MR reduction, while systolic blunting or reversal suggests residual MR or elevated left atrial pressure. Although data in TMVR are limited, parallels from mitral repair show that E/e′ >14 or shortened deceleration time indicate increased filling pressures and may predict limited symptomatic improvement despite technical success [68].

TEE also assesses LVOT dynamics after deployment. A high transmitral gradient with a late-peaking systolic jet into the LVOT suggests dynamic obstruction from AML displacement or bulky inflow. Continuous-wave Doppler documenting a systolic peak > 20 mmHg and visualization of flow acceleration or SAM allow for rapid recognition and management [31,55,69]. Depending on severity, intervention may include repositioning, further expansion, or even the abortion of deployment if feasible.

## 4. Beyond Conventional TEE

Beyond conventional TEE, several advanced imaging modalities have expanded the armamentarium for TMVR guidance. 3D-TEE with multiplanar reconstruction (MPR) provides interactive orthogonal views that enhance annular sizing, trajectory planning, and post-deployment evaluation. Intracardiac echocardiography (ICE) offers high-resolution imaging without general anesthesia, representing an attractive option in high-risk patients or when TEE is contraindicated. Fusion imaging, which integrates 3D TEE with fluoroscopy, projects echocardiographic landmarks directly into the interventional field, improving navigation, coaxial alignment, and device deployment.

Together, these modalities extend the scope of intraprocedural imaging, enhancing procedural precision, safety, and adaptability in complex TMVR cases.

### 4.1. TEE with Multiplanar Reconstruction

3D-TEE with multiplanar reconstruction (MPR) enables simultaneous interactive orthogonal views, typically two long-axis and one short-axis, derived from a single full-volume dataset [6].

This approach allows for precise alignment with the mitral annulus, commissures, and LVOT, supporting accurate annular sizing, trajectory planning, and post-deployment assessment [70].

**Figure 2:**
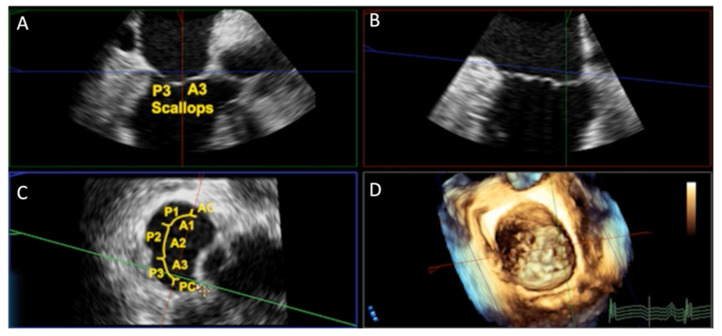
Three-dimensional transesophageal echocardiography (3D TEE) with multiplanar reconstruction (MPR) of the mitral valve. Legend: MPR from 3D TEE displaying orthogonal long- and short-axis views of the mitral valve. (**A**,**B**) Long-axis planes demonstrating leaflet morphology and scallop identification (A3–P3). (**C**) En-face “surgeon’s view” illustrating mitral scallops (A1–A3, P1–P3) and commissures. (**D**) Corresponding 3D volumetric rendering showing the mitral annulus and leaflet configuration. MPR facilitates precise assessment of mitral anatomy, leaflet pathology, and annular geometry, supporting pre-procedural planning and intraprocedural guidance during TMVR.

Compared with conventional 2D imaging, MPR enhances spatial understanding by permitting the real-time rotation and re-slicing of datasets, improving the delineation of leaflet pathology, annular calcification, and prosthesis–tissue interactions [71]. Clinically, this translates into a more accurate recognition of anatomical risk factors such as leaflet overhang, commissural asymmetry, and septal hypertrophy, which directly influence device choice and procedural strategy (Figure 2).

In ViV or ViR procedures, MPR improves the assessment of true internal diameters of rings or surgical prostheses, reducing the risk of under- or over-sizing and better anticipating residual gradients or paravalvular regurgitation [5]. Baseline and post-deployment Doppler interrogation can then be integrated with MPR datasets to quantify transmitral gradients and localize regurgitant jets, enabling targeted corrective maneuvers such as balloon post-dilation or focal closure. The ability to choose between single-beat acquisitions, which minimize stitching artifacts in atrial fibrillation or hemodynamic instability, and multi-beat datasets, which maximize spatial resolution in stable patients, further optimizing imaging quality and reliability.

Early clinical experiences and prospective series in structural interventions confirm that live 3D MPR improves operator confidence, enhances device positioning, and reduces procedural time and contrast use, outcomes that translate directly into safer and more effective TMVR [5,6,70]. In aggregate, 3D TEE with MPR provides the geometric fidelity and real-time adaptability that make it indispensable across all phases of TMVR, from pre-procedural planning to intraprocedural guidance and post-implant evaluation.

### 4.2. Intracardiac Echocardiography

ICE has emerged as a valuable adjunct, and in selected centers, a potential alternative to TEE for TMVR guidance. Its main advantage is the ability to provide high-resolution intracardiac imaging without general anesthesia or endotracheal intubation, which are typically required for prolonged TEE examinations. This makes ICE particularly appealing in high-risk or frail patients, or in those with severe pulmonary disease, or contraindications to esophageal instrumentation [72,73,74]. ICE catheters, usually positioned in the right atrium or advanced into the left atrium via transseptal puncture, can deliver near-field visualization of the interatrial septum and guide transseptal puncture and sheath advancement with excellent spatial resolution [75].

Clinical experience supports its feasibility and safety. In a study of 42 patients undergoing valve-in-valve TMVR under conscious sedation with ICE guidance, outcomes were comparable to those of 14 patients treated with TEE under general anesthesia, with no significant differences in PVL or 30-day mortality. Notably, the ICE group demonstrated a shorter length of hospital stay [76].

Similarly, case series using four-dimensional ICE have demonstrated adequate visualization for device deployment and procedural success under conscious sedation, including in high-risk patients [74,77].

Nonetheless, ICE has limitations. Its smaller field of view and reduced panoramic capability may miss small or eccentric PVL, particularly in complex or heavily calcified anatomies. Although advances in 3D and 4D ICE have markedly improved resolution and color Doppler imaging, image quality and reproducibility remain variable across centers. Catheter stability and manipulation also require significant operator experience.

At present, ICE complements rather than replaces TEE, with the latter remaining the gold standard for comprehensive intraprocedural monitoring. Nonetheless, ongoing technological advances and accumulating clinical data are likely to expand the role of ICE in selected TMVR cases—particularly within minimalist procedural strategies designed to reduce anesthesia exposure and hospital stay [72,73,74].

### 4.3. Fusion Imaging

Fusion imaging is a new technology that combines echocardiographic datasets with live fluoroscopic images, allowing landmarks to be projected directly into the interventional field. By integrating the anatomical detail of 3D TEE with the spatial orientation of fluoroscopy, it enhances real-time navigation, improves the precision of device delivery, and may reduce procedural radiation and contrast burden [78,79,80].

**Figure 3:**
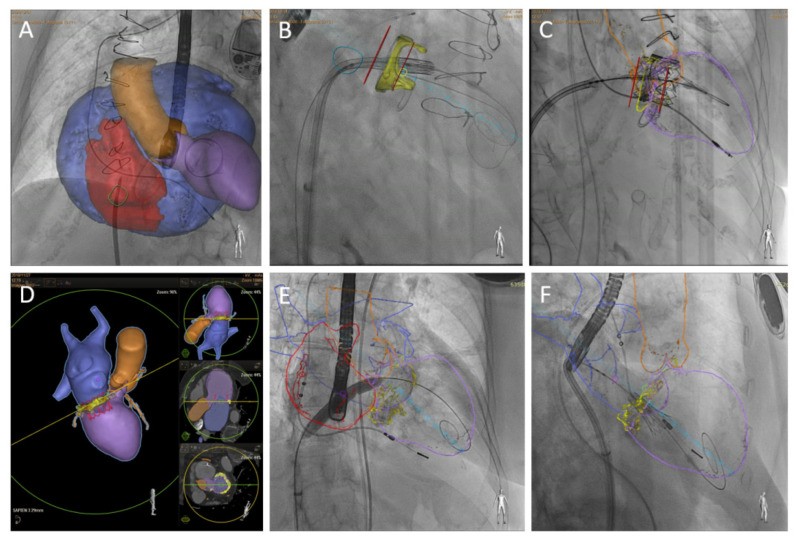
CT-fluoroscopy fusion imaging. The superior row shows a TMVR valve-in-valve procedure in a patient with extreme left atrium enlargement and a modified projection required for transeptal puncture (**A**). Markers (red lines) may be over-imposed on fluoroscopy imaging to guide depth deployment (**B**,**C**). Inferior row, TMVR valve-in-MAC CT preprocedural planning (**D**), interatrial septal balloon dilatation (**E**), and initial phase of THV deployment with coaxial projection to mitral annulus (**F**). Reproduced from ref. [4] published by MDPI, Basel, Switzerland, under the Creative Commons Attribution License (CC BY) [4].

From a technical standpoint, modern fusion platforms (e.g., Philips EchoNavigator, Siemens TrueFusion, GE Valve Assist) synchronize 3D TEE volumes with fluoroscopic orientation by using anatomical markers such as the aortic valve, left atrial appendage, or transseptal puncture site. These systems allow operators to “tag” key structures on TEE, such as the fossa ovalis, mitral annulus, commissures, or LVOT, and project them as overlays onto fluoroscopy. The operator can then manipulate the delivery system under fluoroscopy while continuously visualizing TEE-derived anatomic targets, without repeatedly switching visual attention between separate monitors [81] (Figure 3).

Clinically, fusion imaging is advantageous in several phases of TMVR. During transseptal puncture, it allows for precise confirmation of the puncture height and location by displaying the TEE-defined fossa ovalis directly on fluoroscopy, minimizing needle misdirection [82]. During device navigation, fusion overlays help guide catheters along the intended trajectory toward the mitral inflow, reducing the risk of non-coaxial entry into the annulus. At the stage of valve deployment, fusion markers facilitate verification of final prosthesis alignment, ensuring optimal seating and skirt apposition relative to the annular plane [83].

Although TMVR randomized data are lacking, preliminary data support its procedural value. Fusion imaging is associated with a significant reduction in fluoroscopy time (mean ~12.6 vs. ~18.6 min) and radiation dose in comparison to standard imaging workflows [84]. Another study noted that fusion imaging is associated with enhanced procedural confidence and reduced contrast usage in complex mitral anatomy, although actual numbers in fully TMVR-only cohorts remain limited [85].

## 5. Focus on TEE in Challenging TMVR Scenarios

TEE is indispensable in complex TMVR settings, such as valve-in-valve and valve-in-ring procedures, annuloplasty repairs, interventions for severe mitral annular calcification, and in strategies designed to prevent LVOTO. Beyond routine monitoring, it provides real-time anatomical and functional insights that complement CT and fluoroscopy, enabling precise device sizing, deployment guidance, and complication prevention and management.

### 5.1. Valve-in-Valve, Valve-in-Ring, and Annuloplasty

3D-TEE en-face views allow direct measurement of the true internal diameter of the previous surgical prosthesis, identifying deformation or eccentric geometries that are crucial for accurate sizing and for preventing migration or paravalvular leakage [5,6,80].

TEE can also detect leaflet overhang, a phenomenon in which degenerated bioprosthetic leaflets prolapse into the orifice, reducing the effective valve area and interfering with coaxial seating, particularly relevant for balloon-expandable valves such as Sapien 3 [17,50].

Baseline Doppler is equally relevant, as transmitral gradients of ≥5 mmHg in degenerated prostheses often predict post-implant obstruction [56]. After valve deployment, TEE confirms full frame expansion, detects incomplete apposition or rocking suggestive of undersizing, and maps paravalvular jets, typically mild in valve-in-valve but more pronounced in valve-in-ring cases due to rigid or incomplete ring geometry [86,87]. Importantly, because metallic components can create acoustic shadowing, MPR and off-axis imaging are often required to ensure accurate interpretation [88]. Clinical evidence from the MITRAL registry and other series supports the value of systematic TEE evaluation of leaflet overhang, commissural orientation, and prosthetic geometry, which correlates with lower residual gradients and higher procedural success [64,88]. In this setting, TEE provides not only confirmatory imaging but essential anatomical and functional insights that directly guide device selection, deployment, and long-term outcomes.

### 5.2. Valve-in-MAC (ViMAC)

TMVR in severe MAC represents one of the most challenging structural heart interventions, with high risks of underexpansion, PVL, and LVOTO due to the rigid and irregular annular anatomy [89]. Here, TEE provides unique, real-time insights beyond CT, particularly in evaluating prosthesis–annulus interaction during deployment. Using 3D MPR, operators can measure the neo-LVOT throughout the cardiac cycle and assess anterior leaflet length, mobility, and the aorto–mitral angle, key determinants of LVOTO [20,90].

During valve release, high-frame rate 3D TEE visualizes the skirt expansion against heavily calcified segments, detecting asymmetric frame distortion or eccentric gaps not visible on fluoroscopy. Serial en-face datasets help identify subtle device rocking caused by calcium spurs. TEE is also essential for detecting PVL: commissural calcium bridges and nodular protrusions are typical sites of jet formation, and 3D color Doppler with vena contracta area mapping helps discriminate focal leaks (amenable to post-dilation or plug closure) from diffuse leaks due to incomplete seating.

Advanced Doppler assessment complements these findings. Continuous-wave Doppler across the LVOT reveals early obstruction by dagger-shaped, late peaking signals, while simultaneous transmitral and LVOT interrogation distinguishes obstruction from underexpansion. Pulmonary venous Doppler adds sensitivity, as new systolic blunting or reversal often precedes hemodynamic instability.

Clinical data from the MITRAL trial and subsequent registries confirm that intraprocedural TEE detection of skirt malapposition, eccentric PVL, and evolving LVOTO predicts procedural outcomes and early mortality [91]. The Tendyne TVMR system, recently FDA-approved (May 2025) for patients with severe MAC ineligible for surgery or TEER, is fully repositionable and self-expanding, designed to meet the challenges of calcified, irregular annuli.

This milestone underscores that high-resolution 3D TEE is indispensable for real-time guidance, ensuring optimal positioning, full expansion, and early complication detection in these complex anatomies.

### 5.3. LVOTO Prevention

Among emerging techniques to prevent LVOTO during TMVR, TEE plays a pivotal role in the guidance of procedures such as LAMPOON, BATMAN, and SESAME [92]. In the LAMPOON (Laceration of the Anterior Mitral Leaflet to Prevent Outflow Obstruction) procedure, TEE is indispensable from patient selection to intraprocedural monitoring [93]. Multiplanar and 3D views confirm traversal of the basal A2 segment, avoiding chordal or papillary entanglement, and visualize progressive leaflet laceration while excluding injury to adjacent structures [94]. After laceration and valve implantation, color and continuous-wave Doppler quantify transmitral gradients, verify laminar LVOT flow, and document restoration of a safe neo-LVOT area > 200 mm^2^ [92,95].

In the pivotal NIH trial, TEE-guided LAMPOON achieved 100% procedural success in high-risk patients, validating the threshold of neo-LVOT < 170–200 mm^2^ as predictive of obstruction and confirming that LAMPOON can effectively enlarge the outflow area beyond the safety limit [93]. These results underscore the value of integrating quantitative TEE measurements into procedural planning.

**Figure 4:**
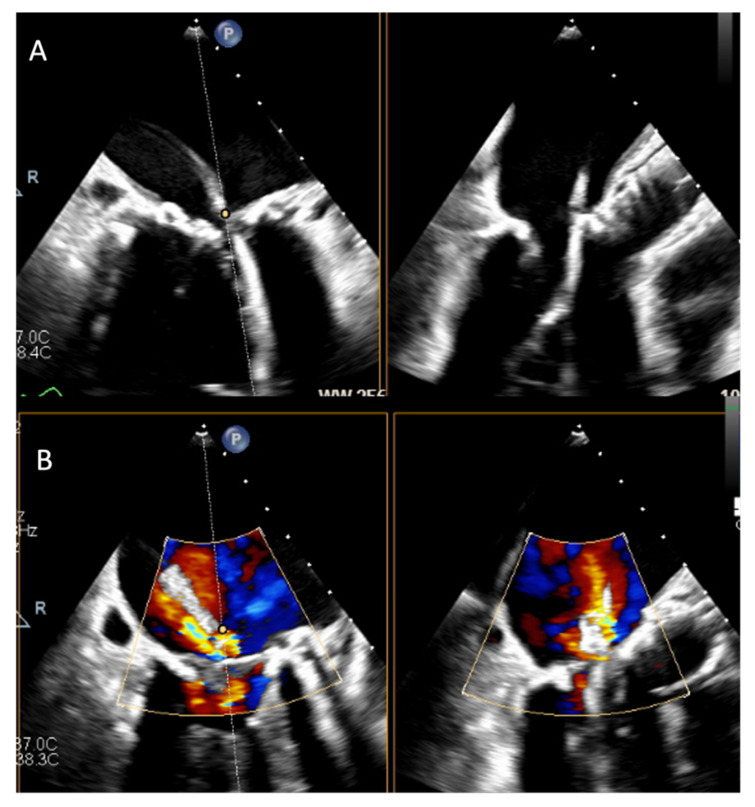
Procedural steps for the BATMAN procedure. (**A**) Positioning of the catheter across the anterior mitral leaflet. (**B**) Positioning of the balloon across the mitral leaflet.

In contrast, the BATMAN (Balloon Assisted Translocation of the Mitral Anterior Leaflet) technique displaces rather than lacerates the AML. Pre-procedurally, TEE identifies anatomical risk factors such as leaflet length or septal bulge. During the procedure, 3D MPR ensures correct balloon positioning at the leaflet base without chordal entanglement, and inflation is monitored in real-time to verify leaflet displacement away from the LVOT [96,97] (Figure 4).

After valve implantation, TEE confirms stable prosthesis seating, absence of dynamic LVOTO, and a neo-LVOT >200 mm^2^ [50]. Early series demonstrate feasibility and effective LVOT gradient relief, suggesting BATMAN as a potential alternative to LAMPOON in selected anatomies [98].

The ROBIN (Retrograde Balloon-Induced Anterior Mitral leaflet laceration) technique represents a modification of the LAMPOON for valve-in-valve or valve-in-ring TMVR cases at high risk of neo-LVOTO. TEE guides both the pre-procedural evaluation of leaflet anatomy and intra-procedural visualization of controlled electrosurgical leaflet laceration, confirming expansion of the neo-LVOT and exclusion of residual obstruction or PVL [99,100].

Finally, SESAME (Septal Scoring Along the Midline Endocardium) targets the septum rather than the anterior leaflet to mitigate obstruction. Pre-procedurally, TEE, often combined with CT, assesses septal thickness (>15 mm), the aorto–mitral angle, and predicted neo-LVOT dimensions. During intervention, TEE guides catheter positioning, monitors septal scoring, and ensures that the incisions remain confined to the endocardium.

Color Doppler confirms relief of flow acceleration, while continuous-wave Doppler excludes new gradients or perforation. After valve deployment, 3D TEE verifies an enlarged neo-LVOT (>200 mm^2^), coaxial alignment, and transmitral gradients <5 mmHg [101]. Although still early in development, initial reports indicate SESAME can be safely performed under TEE guidance, potentially broadening TMVR eligibility for anatomically complex patients [102].

## 6. TEE in Recent Guidelines and Consensus for TMVR

Recent international guidelines and expert consensus statements consistently underscore the central role of echocardiography, particularly TEE, across the TMVR pathway, while emphasizing its complementarity with CT. The 2025 ESC/EACTS Guidelines identify CT as the reference standard for annular sizing, neo-LVOT prediction, and device modeling, whereas TEE is identified as the essential tool for intra-procedural guidance, including transseptal puncture, device navigation, coaxial alignment, and immediate hemodynamic assessment [103].

The earlier 2021 ESC/EACTS guidelines similarly highlight the central role of 2D and 3D TEE throughout the TMVR for accurate patient selection, grading of valvular dysfunction, and post-implant surveillance of transvalvular gradients and residual regurgitation [104]. The 2020 ACC/AHA Guidelines also reinforce a multimodality imaging approach, positioning TEE as the cornerstone of intraprocedural monitoring within structural heart programs [105]. In parallel, the AATS/ACC/SCAI/STS consensus statement endorses systematic TEE use throughout all phases of transcatheter mitral procedures [106].

From a technical standpoint, the ASE/EACVI recommendations advocate for 3D TEE with MPR in annular quantification, device sizing, and PVL detection. Doppler assessment remains key for confirming procedural success, notably achieving a mean transmitral gradient <5 mmHg at controlled heart rate [55,107].

In synthesis, current guidelines converge on a multimodality imaging paradigm: CT as the gold standard for pre-procedural planning and sizing, and TEE as the indispensable real-time modality for procedural execution and immediate outcome assessment, both integrated within standardized workflows and multidisciplinary Heart Team decision-making.

## 7. Integrated Best Practices and Clinical Takeaways for TMVR Imaging

Optimizing workflows for TMVR requires close collaboration among imaging specialists, interventional cardiologists, cardiac surgeons, anesthesiologists, and heart failure experts within a cohesive Heart Team. Standardized imaging protocols should integrate the TEE and CT datasets, reviewed during dedicated meetings to reach consensus on patient selection, device choice, and procedural strategy.

Clear task allocation, such as assigning one echocardiographer to real-time imaging and another to interpretation, reduces errors and strengthens communication. Structured post-procedural reports summarizing TEE findings enable the early detection of complications and coordinated follow-up.

Embedding TMVR-specific checklists into routine practice, including pre-procedural (annular sizing, neo-LVOT prediction, interatrial septum evaluation), intraprocedural (puncture site, device alignment, paravalvular leak screening), and post-implant (gradients, LVOT dynamics, pericardial effusion) checkpoints, promotes reproducibility and minimizes operator variability.

Regular Heart Team reviews promote continuous improvement, while emerging tools such as fusion imaging, AI-based quantification, and virtual reality planning can be progressively integrated into this framework.

Within this workflow, TEE remains the key imaging modality that translates TMVR planning into procedural success. Pre-procedurally, it complements CT by confirming annular dimensions, LVOT geometry, and chamber function, thereby preventing oversizing, malposition, or obstruction. Intraprocedurally, 3D TEE with multiplanar reconstruction enables en face annular visualization, precise transseptal puncture guidance, and continuous tracking of device trajectory.

After deployment, Doppler interrogation provides immediate feedback on transmitral gradients, paravalvular sealing, and LVOT dynamics, allowing corrective maneuvers. In anatomically complex scenarios, TEE uniquely identifies anchoring issues, leaflet overhang, and commissural alignment, offering insights beyond the scope of fluoroscopy alone and ensuring device stability and hemodynamic competence.

Consistent adherence to these principles has been associated with fewer complications and improved procedural outcomes. Although ICE and fusion imaging are expanding the field, standardized TEE protocols and operator expertise remain the cornerstone of TMVR imaging.

## 8. Conclusions

TEE remains the cornerstone of intraprocedural imaging in TMVR, providing real-time anatomical and hemodynamic guidance for device implantation that complements CT planning and directly impacts procedural success. Its role is particularly critical in complex anatomies, where systematic protocols and Heart Team integration ensure safety and reproducibility despite inherent limitations. Emerging technologies such as AI, fusion imaging, and virtual platforms are expected to complement rather than replace TEE, confirming its place within innovative, standardized multimodality workflows that expand eligibility and improve outcomes.

## Figures and Tables

**Table 1:** Annular sizing ranges and device-specific considerations in TMVR.

Device		Manufacturer	Type	Annular Sizing Range	Key Considerations
Tendyne	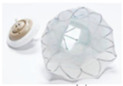	Abbott (Abbott Park, IL, USA)	Self-expanding, tethered	Broad range, up to ~50 mm annular diameter	Largest size matrix; suitable for large annuli; less ideal for very small annuli
Intrepid	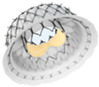	Medtronic (Dublin, Ireland)	Self-expanding, dual-stent	Restricted to ~36–43 mm outer frame diameters	Anchors in annulus/leaflets; limited for very small or large annuli
Sapien 3 (ViMAC, ViV, ViR)	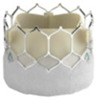	Edwards Lifesciences (Irvine, CA, USA)	Balloon-expandable	Up to ~29 mm outer diameter	Widely used in ViV/ViR/ViMAC; risk of oversizing in small annuli; may be inadequate for large annuli
HighLife	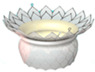	HighLife SAS (Paris, France)	Two-component system (subannular ring + prosthesis)	Limited published ranges; typical native annuli	Novel anchoring system; investigational; sizing less standardized
Altavalve	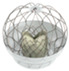	4C Medical (Maple Grove, MN, USA)	Self-expanding, atrial anchoring	Early feasibility data; ~27–51 mm annular diameters	Investigational; designed for broad annular compatibility; ongoing evaluation

Legend: ViV: Valve-in-Valve, ViR: Valve-in-Ring, ViMAC: Valve-in-Mitral annulus Calcification.

**Table 2:** Comparative TMVR Echocardiographic Cut-offs.

Parameter	Cut-Off/Threshold	Clinical Implication
Predicted neo-LVOT area	<200 mm^2^ = high risk of LVOTO	Values below threshold predict severe LVOTO, often requiring adjunctive strategies (LAMPOON, BATMAN, SESAME).
Anterior Mitral Leaflet length	>25 mm associated with increased obstruction risk	Excessive leaflet length predisposes to systolic anterior motion and LVOT encroachment.
LVEF	<30–35% = impaired prognosis	Reduced LVEF predicts limited procedural benefit and higher perioperative risk.
TAPSE	<17 mm indicates RV dysfunction	Reduced TAPSE reflects RV dysfunction, associated with worse survival post-TMVR.
Transmitral mean gradient (post-TMVR)	<5 mmHg at HR ~70 bpm considered acceptable	Elevated gradients suggest device underexpansion, malalignment, or prosthesis-patient mismatch.

Legend: LVOT: Left Ventricular Outflow Tract, LVOTO: Left Ventricular Outflow Tract Obstruction, TAPSE: Tricuspid Annular Plane Systolic Excursion, RV: right ventricle, HR: heart rate, TMVR: Transcatheter mitral valve replacement, LAMPOON: Laceration of the Anterior Mitral Leaflet to Prevent Outflow Obstruction, BATMAN: Balloon-Assisted Translocation of the Mitral Anterior Leaflet, SESAME: Septal Scoring Along the Midline Endocardium.

## Data Availability

No new data were created or analyzed in this study. Data sharing is not applicable to this article.

## Abbreviations

The following abbreviations are used in this manuscript:

AML	Anterior Mitral Leaflet
AP	Anteroposterior
BATMAN	Balloon-Assisted Translocation of the Mitral Anterior Leaflet
CT	Computed Tomography
EACVI	European Association of Cardiovascular Imaging
ICE	Intracardiac Echocardiography
IVS	Interventricular Septum
LA	Left Atrium
LAMPOON	Laceration of the Anterior Mitral Leaflet to Prevent Outflow Obstruction
LV	Left Ventricle
LVEF	Left Ventricular Ejection Fraction
LVOT	Left Ventricular Outflow Tract
LVOTO	Left Ventricular Outflow Tract Obstruction
MAC	Mitral Annular Calcification
MPR	Multiplanar Reconstruction
MR	Mitral Regurgitation
MV	Mitral Valve
PVL	Paravalvular Leak
ROBIN	Retrograde Balloon-Induced Anterior Mitral leaflet laceration
RV	Right Ventricle
SAM	Systolic Anterior Motion
SESAME	Septal Scoring Along the Midline Endocardium
S′	Peak Systolic Velocity
TAPSE	Tricuspid Annular Plane Systolic Excursion
TEE	Transesophageal Echocardiography
TEER	Transcatheter Edge-to-Edge Repair
TMVR	Transcatheter Mitral Valve Replacement
ViMAC	Valve-in-Mitral Annular Calcification
ViR	Valve-in-Ring
ViV	Valve-in-Valve
